# Physicochemical, structure properties and *in vitro* hypoglycemic activity of soluble dietary fiber from adlay (*Coix lachryma-jobi* L. var. *ma-yuen* Stapf) bran treated by steam explosion

**DOI:** 10.3389/fnut.2023.1124012

**Published:** 2023-02-03

**Authors:** Xinjing Tang, Zhirong Wang, Jiong Zheng, Jianquan Kan, Guangjing Chen, Muying Du

**Affiliations:** ^1^College of Food Science, Southwest University, Chongqing, China; ^2^Chinese-Hungarian Cooperative Research Centre for Food Science, Chongqing, China; ^3^Chongqing Key Laboratory of Speciality Food Co-Built by Sichuan and Chongqing, Chongqing, China; ^4^College of Food and Pharmaceutical Engineering Institute, Guiyang University, Guiyang, Guizhou, China

**Keywords:** adlay bran, soluble dietary fiber, steam explosion, physicochemical properties, structural characteristics, *in vitro* hypoglycemic activity

## Abstract

To enhance the content of adlay bran soluble dietary fiber (SDF) and improve its functionality, we investigated the influences of steam explosion (SE) on the physicochemical, structural properties, and *in vitro* hypoglycemic activities of adlay bran SDF. The cellulose, hemicellulose, and lignin contents of adlay bran decreased significantly after SE treatment. When the SE strength was 0.8 MPa for 3 min, the SDF content was 9.37%, which was a significant increase of 27.48% compared to the control. Under these conditions, SDF showed the highest oil-holding capacity (OHC) (2.18 g/g), cholesterol adsorption capacity (CAC) (27.29 mg/g), glucose adsorption capacity (GAC) (15.54 mg/g), glucose dialysis retardation index (GDRI) (36.57%), and α-Amylase activity inhibition ratio (α-AAIR) (74.14%). Compared with SDF from untreated adlay bran, SDF from SE-treated adlay bran showed lower weight molecular. In addition, differential scanning calorimetry (DSC) measurement showed that the peak temperature of SDF from adlay bran treated by SE increased by 4.19°C compared to the untreated SDF sample. The structure of SDF from adlay bran treated by SE showed that the SDF surface was rough and poriferous and the specific surface areas increased. In conclusion, SE pretreatment increases the content of SDF in adlay bran and improves its physicochemical, structural properties, and biological activities, which will be beneficial for the further exploitation of adlay bran.

## 1. Introduction

Adlay (*Coix lachryma-jobi* L. var. *ma-yuen* Stapf) is a functional food in Asia that is also called Job’s tears and which has been proven to exhibit various anti-inflammatory (1), anti-cancer (2), and anti-allergic effects (3). Therefore, adlay is widely utilized and processed into a variety of products, and the processing of these products generates large amounts of adlay bran. Adlay bran, a primary by-product of adlay processing, is rich in a broad spectrum of nutrients, including phenolic, polysaccharides, fatty acids and dietary fiber (DF), all of which exhibit certain bioactivities, including anti-hemolysis, anti-proliferative, and xanthine oxidase inhibitory capacities (4, 5). However, adlay bran is typically used as feed or discarded as waste. This phenomenon results in a huge waste of resources and greatly reduces the overall economic value of adlay. In recent years, DF has received increasing attention in production applications of food industry due to its multiple health-promoting benefits. DF is a carbohydrate-based polymer whose intake is associated with overall metabolic health and which has been proposed to ameliorate a variety of other pathologies, including cardiovascular disease, colon dysfunction, impaired gut motility and colorectal carcinoma risk (6, 7). Not only does DF help regulate intestinal function, but it also increases stool volume and maintains stable postprandial blood glucose (8, 9). Based on its water solubility, DF is usually classified into soluble dietary fiber (SDF) and insoluble dietary fiber (IDF) (10). It is well-documented that SDF is primarily responsible for the bioactivities of DF, based on its physiological functions and physicochemical properties (11). For example, SDF showed stronger anti-hyperlipidemia activity than IDF, and it plays a major role in postprandial glucose and insulin response (12, 13). In addition, SDF has antioxidant activity, oil-holding capacity (OHC) and cholesterol-lowering capacity (14, 15). Adlay bran has the potential to be used as a source of DF; in particular SDF from adlay bran is similar in composition to adlay, endowing the adlay-bran-based products with additional functional properties. However, the low levels of SDF in natural DF and adlay bran limits food applications. Therefore, appropriate methods to increase the SDF content can make it more widely used in the food industry.

Currently, methods of DF modification have been reported, including physical (16), chemical (17), and biological methods (18). Among these, physical methods, which are used most commonly, have the advantages of high efficiency and environmental protection. Steam explosion (SE) is an emerging physical modification technique which mainly used in the treatment of wood fibers. During SE, a sample is rapidly heated by high-pressure water vapor for a given time, after which a rapid release of pressure causes the steam to expand within the sample, resulting in the separation of individual fibers and destruction of the raw material structures (19). Under these circumstances, the effects of acidolysis, mechanical breakage and thermal degradation can degrade and convert cellulose and hemicellulose into SDF. In addition, compared with other pretreatment methods, SE enables faster mass transfer, has a lower energy cost, requires less hazardous process chemicals, and involves simple technical manipulation (20, 21). In recent years, SE has also been used to modify DF, including improving the content of SDF from sweet potato residue (22), wheat bran (23), and okara (24). However, the application of SE in SDF extraction from adlay bran has not yet been reported, and the effects of SE on the physicochemical, structure properties and *in vitro* hypoglycemic activity of SDF from adlay bran are unclear.

Based on the aforementioned points, the objective of this present study was to investigate the influences of SE modification on the physicochemical, structure properties, and *in vitro* hypoglycemic activity of adlay bran SDF. To these ends, the adlay bran was treated by SE at different conditions. Then, SDF was extracted from adlay bran by enzymatic-hydrolysis method. Finally, the physicochemical properties [e.g., OHC, cholesterol adsorption capacity (CAC)] and in vitro hypoglycemic activity [e.g., glucose adsorption capacity (GAC), α-Amylase activity inhibition ratio (α-AAIR), glucose dialysis retardation index (GDRI)] of SDF before and after SE-treatment were determined and evaluated. In addition, scanning election microscopy (SEM), differential scanning calorimetry (DSC) and Fourier transform infrared spectroscopy (FT-IR) were also used to evaluate the effects of SE on the structure of SDF.

## 2. Materials and methods

### 2.1. Materials and chemicals

Adlay bran was purchased from a local rural supplier (Guizhou, China) and stored at 4°C until use. Cellulose reagent kit, hemicellulose reagent kit and lignin reagent kit were obtained from Suzhou Michy Biomedical Technology Co., Ltd. (Suzhou, China). Thermostable α-amylase (2,600 U/g) was supplied by Shanghai Aladdin Biochemical Technology (Shanghai, China). Alkaline protease (200 U/mg) and cholesterol standard were obtained from Beijing Solarbio Technology (Beijing, China). 3, 5-dinitrosalicylic acid was purchased from Guangzhou Caozhiyuan Biotechnology (Guangzhou, China). Glucose standard was purchased from Tianjin Solomon Biotechnology (Tianjin, China). Other chemical reagents were of analytical grade.

### 2.2. Steam explosion treatment

Based on preliminary laboratory studies, steam pressures of 0.8, 1.2, and 1.6 MPa were selected, and residence times were set at 1, 2, and 3 min. Approximately 500 g of adlay bran was loaded into the 2 L reactor of a SE system (QB-300, Zhang dao Co., Ltd., Hebi, China), and high-pressure steam was introduced into the reactor chamber. After the steam pressure has been maintained for a given time, and then pressure was released immediately. The adlay bran was collected after SE, dried in a hot blast oven (GZX-9140 MBE, Shanghai, China) at 50°C and crushed through a 60-mesh sieve. Crushed bran was then defatted by Soxhlet extraction using petroleum ether (boiling range 30–60°C) at 70°C for 10 h. Residual solvent was removed by hot air drying at 60°C, after which samples were stored at 4°C.

### 2.3. Extraction of adlay bran SDF and calculation of yield

Soluble dietary fiber was prepared using the enzymatic-hydrolysis method (21) with slight modifications. The pretreatment bran power was dispersed in 25 times volumes of phosphate buffer solution (pH 6.5), containing 100 μL thermostable α-amylase, and this solution was subjected to further hydrolysis at 98°C for 30 min. The hydrolysate was then cooled to 50°C and adjusted to a pH of 10.0 using 6 M NaOH solution. Subsequently, 80 μL of alkaline protease was added, and samples were subjected to further hydrolysis for 2 h. After enzymatic hydrolysis reaction, the hydrolysate was adjusted to a pH of 4.5 with 6 M HCI solution to inactivate enzymes. The supernatant was obtained by centrifugation at 4,000 × *g* for 10 min and evaporated to approximately one-tenth volume using rotary evaporation (RE-52, Shanghai, China). Subsequently, the concentrated supernatant was added into 95% (v/v) ethanol solution, incubated at 25°C for 12 h. Finally, the precipitate was obtained by centrifugation and dried at 60°C. The dried precipitate crushed, sifted through a 60-mesh sieve, and then stored at 4°C, the power obtained was SDF. The SDF yield was calculated as follows:


$$\mathrm{SDF}\,\mathrm{yield}\,\left(100\%\right)=\left(W_{1}/W\right)\times 100$$

Where W_1_ is the weight of SDF obtained after drying (g); W is the weight of adlay bran sample (g).

### 2.4. Chemical composition assays

Levels of cellulose, hemicellulose, and lignin from adlay bran before and after SE pretreatment were assessed by the spectrophotometric method according to kit instructions.

### 2.5. Physicochemical properties of SDF from adlay bran

#### 2.5.1. Oil-holding capacity (OHC)

The OHC was performed according to the method described by Wang et al. (21). A total of 0.5 g of SDF was mixed with 10 mL olive oil in a centrifugal tube and placed at 4°C for 1 h. Then the mixture was centrifuged at 4,200 × *g* for 15 min, the supernatant was removed and the residue was weighed. The OHC was calculated as follows:


$$\text{OHC}\,\left(g/g\right)=\left({\text{W}}_{1}-W\right)/W$$

Where W_1_ is the weights of SDF sample after oil absorption (g); W is the weight of SDF sample (g).

#### 2.5.2. Cholesterol adsorption capacity (CAC)

The CAC was performed using the method described by Chen et al. (25) with slight modifications. Fresh egg yolk was whipped with nine volumes of hot distilled water. Accurately weigh 500 mg of SDF into 15 mL of diluted yolk emulsion. After that, the mixture was adjusted to a pH of 7.0 using NaOH solution, incubated at 37°C for 120 min, and then centrifuged. The supernatant was obtained for determination of cholesterol concentration by o-phthalaldehyde method. The CAC was calculated as follows:


$$\mathrm{CAC}\,\left(\mathrm{mg}/g\right)=\left({\text{C}}_{\text{i}}-{\text{C}}_{\text{s}}\right)\times V/W$$

Where C_i_ is the concentration of cholesterol in the diluted yolk emulsion; C_s_ is the concentration of cholesterol in the diluted yolk emulsion after SDF adsorption (mg/mL); W is the weight of SDF (g), and V is the volume of diluted yolk (mL).

### 2.6. *In vitro* hypoglycemic activity of SDF

#### 2.6.1. Glucose adsorption capacity (GAC)

The GAC was determined according to the method by Ma and Mu (26) with slight modifications. Accurately weigh 1 g SDF into 100 mL of glucose solution (100 mmol/L). After incubation for 6 h at 37°C in an incubator, the supernatant was obtained by centrifugation. Finally, the concentration of glucose in the supernatant was measured using 3, 5- Dinitrosalicylic acid method. The GAC was calculated as follows:


$$\text{GAC}\,\left(\mathrm{mg}/g\right)=\left({\text{C}}_{\text{i}}-{\text{C}}_{\text{s}}\right)\times {\text{V}}_{\text{i}}/W$$

Where W and V_i_ are the weight of SDF (g) and glucose solution volume (mL), respectively; C_i_ and C_s_ are the concentration of glucose in the supernatant before and after SDF adsorption (mg/mL), respectively.

#### 2.6.2. α-Amylase activity inhibition ratio (α-AAIR)

The α-AAIR was evaluated according to the method described by Chau et al. (27). Briefly, 4 mg of α-amylase and 1 g of SDF were added into 40 mL of 4% starch solution, and the mixture was homogenized and oscillated at 37°C for 1 h. Then the mixture was centrifuged, the concentration of glucose in the supernatant was measured using 3, 5- Dinitrosalicylic acid method. α-AAIR was calculated as follows:


$$\alpha -\text{AAIR}\left(\%\right)=\left({\text{C}}_{\text{c}}-{\text{C}}_{\text{s}}\right)\times 100/{\text{C}}_{\text{c}}$$

Where C_c_ and C_s_ are the concentration of glucose in the supernatant with and without SDF (mg/mL), respectively.

#### 2.6.3. Glucose dialysis retardation index (GDRI)

The GDRI was determined using the method described by Tang et al. (28). Briefly, 500 mg of SDF was thoroughly hydrated with 15 mL of glucose solution (100 mmol/L), and then the solution was transferred to a 15 cm dialysis bag. The dialysis bag was put in a beaker containing 200 mL distilled water and incubated at 37°C for 2 h. Finally, at 0.5 h intervals the concentration of glucose in the dialysate was measured using 3, 5- Dinitrosalicylic acid method. The GDRI was calculated as follows:


$$\text{GDRI}\left(\%\right)=\left({\text{C}}_{\text{c}}-{\text{C}}_{\text{s}}\right)\times 100/{\text{C}}_{\text{c}}$$

Where C_s_ and C_c_ are the concentration of glucose in the dialysates with and without SDF, respectively.

### 2.7. Structural characteristics

#### 2.7.1. Particle size distribution

The particle size distributions of SDF were measured with a laser particle size instrument (Mastersizer 3000, Malvern Instruments Co., Ltd., UK). Ethanol was used as a dispersant, the appropriate amount of SDF was taken and ultrasonically assisted to disperse it evenly. The particle size determination range was from 0.01 to 2,000 μm.

#### 2.7.2. Scanning election microscopy (SEM)

The microstructure of SDF from adlay bran before and after SE pretreatment was obtained using a SEM (Phenom Pro, Phenom World, Holland). The dried SDF sample was fixed on double-sided conducting adhesive tapes and sputter-coated with gold (29). Subsequently, samples were observed using SEM at 10 kV.

#### 2.7.3. Fourier transform infrared spectroscopy (FT-IR)

Each SDF sample was ground into power and analyzed by spectrum100 FTIR spectrometer (Spectrum 100,9PerkinElmore, USA). IR spectra of SDF samples were recorded from 4,000 to 600 cm^–1^ with a resolution of 4 cm^–1^.

#### 2.7.4. Molecular weight distribution

The molecular weight of SDF was analyzed by gel permeation chromatography (GPC). Samples (10 mg) of SDF from untreated and SE-treated adlay bran were dissolved in 1 mL ultrapure water. Then, the mixture was filtered by 0.45 μm membranes. The chromatographic conditions were as follows: gel chromatography column was TSKgel GMPWXL (TOSOH CORP., Japan), mobile phase was 0.1 mol/L NaCO_3_ at a flow rate of 0.6 mL/min, and injection volume was 100 μL.

#### 2.7.5. Monosaccharide composition

Soluble dietary fiber sample and 2 ml/L trifluoroacetic acid (3 mL) were added to 10 mL ampoules, hydrolyzed at 120°C for 4 h. After removing the trifluoroacetic acid with methanol, the residue was dissolved in 3 mL of water to obtain the polysaccharide hydrolysate. Then, 250 μL of hydrolysate was derivatized with 250 μL 0.6 mol/L NaOH solution and 500 μL 0.4 mol/L PMP-methanol solution for 1 h at 70°C. After cooling to room temperature, 500 μL of 0.3 mol/L HCl was added to neutralize the reaction products. The mixture was centrifuged for 10 min to obtain the supernatant. Then, 1 mL of chloroform was added to the supernatant and the extraction was repeated three times. The analysis conditions were as follows: chromatographic column: Xtimate (C18) column (250 mm × 4.6 mm, 5 μm), column temperature: 30°C, flow rate: 1.0 mL/min and injection volume was 20 μL.

#### 2.7.6. Thermal stability analysis

Thermal analysis of SDF samples from untreated and SE-pretreated adlay bran were analyzed with a differential scanning calorimeter (DSC25, TA Instruments Co., Ltd., USA). Approximately 15 mg of SDF sample was put in a crucible and an empty crucible was used as a control. The crucibles were heated from 30 to 300°C at 50°C/min with a nitrogen atmosphere flow of 50 mL/min.

### 2.8. Statistical analysis

Each experiment was repeated at least in triplicate. The data was subjected to analysis of variance testing using SPSS 26.0 software. The data were expressed as mean ± standard deviation (X ± SD). Differences were considered statistically significant when *p* < 0.05 according to Duncan’s multiple range tests.

## 3. Results and discussion

### 3.1. Effects of steam pressure and residence time on SDF content

**FIGURE 1:**
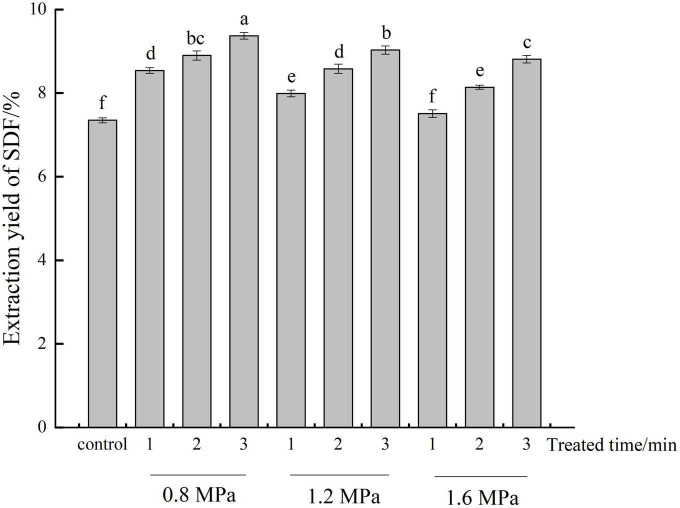
Extraction yields of soluble dietary fiber (SDF) at different steam explosion (SE) treatment conditions. Control: untreated adlay bran; adlay bran treated by SE at different steam pressures (0.8, 1.2, and 1.6 MPa) and different residence times (1, 2, and 3 min). Different lowercase letters over column indicate significant differences (*p* < 0.05).

The influence of different steam pressure and residence time on the content of SDF from adlay bran was presented in Figure 1. SE treatment (except 1.6 MPa, 1 min) significantly improved (*p* < 0.05) the content of SDF by contrast with the untreated control. Moreover, the SDF content generally increased with steam pressure decreased and residence time increased. The highest yield was 9.37% at 0.8 MPa for 3 min. This was significantly increased by 27.48% compared to the control (7.35%). This increase in SDF content is due to the fact that, during SE, material is rapidly swollen by superheated liquid for a given time, the steam into the tissue, after which the pressure is instantly released such that the steam volume abruptly expands (19). Mechanical action causes some fibers breakage and disrupts cell wall structures, which may facilitate dissolution of inclusive SDF and also increase SDF content.

The decrease in extraction yield at pressures beyond 0.8 MPa may be due to high pressure causes cellulose and lignin in the raw material to degrade into smaller molecules which are not precipitated with alcohol, resulting in a lower extraction rate of SDF (21). Shen et al. (30) reported that high SE intensity can cause cellulose, hemicellulose, and other substances to degrade into monosaccharides or oligosaccharides with smaller molecular weights, leading to decreased SDF content in soybean hull.

The SE reaction process includes two parts: pressurized high-temperature cooking and instantaneous release of high pressure. An increase in the residence time essentially extends the pressurized high-temperature cooking time; because to a certain extent, IDF are degraded and converted into SDF with the increase of residence time, the content of SDF keeps increasing (19). It was conclude that the SDF content from adlay bran can be increased by SE pretreatment, and optimized SDF extraction rates can be achieved using a suitable range of steam pressures and residence times.

### 3.2. Chemical compositions

Adlay bran contains a lot of cellulose, hemicellulose, and lignin. These components are interconnected by chemical bonds and hydrogen bonds, making them difficult to separate (31). The instantaneous mechanical action of SE, high pressure and the rapid expansion of water vapor can break the connections between cellulose, hemicellulose, and lignin, and promoting the breakage of glycosidic bonds in large molecules, thus increasing SDF content (32). The cellulose, hemicellulose, and lignin contents of adlay bran pretreated under different steam explosion intensity treatments were determined as shown in Table 1. By contrast with the untreated control, the content of cellulose, hemicellulose and lignin in adlay bran decreased significantly after SE treatment. To be precise, the contents of the three compounds in the adlay bran sample after SE pretreatment at 1.6 MPa for 3 min were reduced by 40.44, 9.65, and 34.95%, respectively, in comparison with the control. This is consistent with other the findings of reduced cellulose, hemicellulose and lignin contents in steam-exploded materials (31). Tanpicha et al. (32) concluded that the SE technique can partially eliminate hemicellulose and lignin from pineapple leaves. Nie et al. (33) concluded that hemicellulose in corn stover was partially degraded during SE treatment. Zhao et al. (34) found that the cellulose and hemicellulose contents of sorghum decreased after SE treatment.

**TABLE 1:** Effect of steam explosion (SE) pretreatment on chemical compositions of adlay bran.

Sample	Cellulose (g/100g)	Hemicellulose (g/100g)	Lignin (g/100g)
Control	24.31 ± 0.08^a^	17.93 ± 0.04^a^	8.44 ± 0.09^a^
0.8 MPa, 1 min	17.17 ± 0.10^d^	16.94 ± 0.03^d^	8.02 ± 0.03^b^
0.8 MPa, 2 min	16.65 ± 0.13^e^	17.17 ± 0.04^c^	6.69 ± 0.10^d^
0.8 MPa, 3 min	18.42 ± 0.10^c^	16.85 ± 0.02^e^	5.74 ± 0.08^h^
1.2 MPa, 1 min	20.49 ± 0.06^b^	16.66 ± 0.03^g^	6.36 ± 0.18^f^
1.2 MPa, 2 min	18.54 ± 0.06^c^	17.00 ± 0.05^d^	5.85 ± 0.03^g^
1.2 MPa, 3 min	16.26 ± 0.25^f^	17.54 ± 0.02^b^	6.56 ± 0.03^e^
1.6 MPa, 1 min	18.70 ± 0.24^c^	16.76 ± 0.06^f^	7.99 ± 0.03^b^
1,6 MPa, 2 min	16.70 ± 0.04^e^	16.74 ± 0.03^fg^	7.57 ± 0.02^c^
1.6 MPa, 3 min	14.48 ± 0.12^g^	16.20 ± 0.05^h^	5.49 ± 0.12^i^

Data represent the mean and standard deviation of each sample assayed in triplicate. Different lowercase letters in the same column indicate significant differences (*p* < 0.05). Control: untreated adlay bran; adlay bran treated by SE at different steam pressures (0.8, 1.2 and 1.6 MPa) and different residence times (1, 2, and 3 min).

The degradation of cellulose and hemicellulose may be due to the fact that glycosidic and hydrogen bonds in the fibers are broken by SE treatment, causing them to form small molecules of reducing sugars to be leached (35). After SE treatment, cellulose, hemicellulose, and lignin are partially degraded into small molecules, thus increasing the SDF content.

### 3.3. Physicochemical properties of SDF

#### 3.3.1. OHC

**FIGURE 2:**
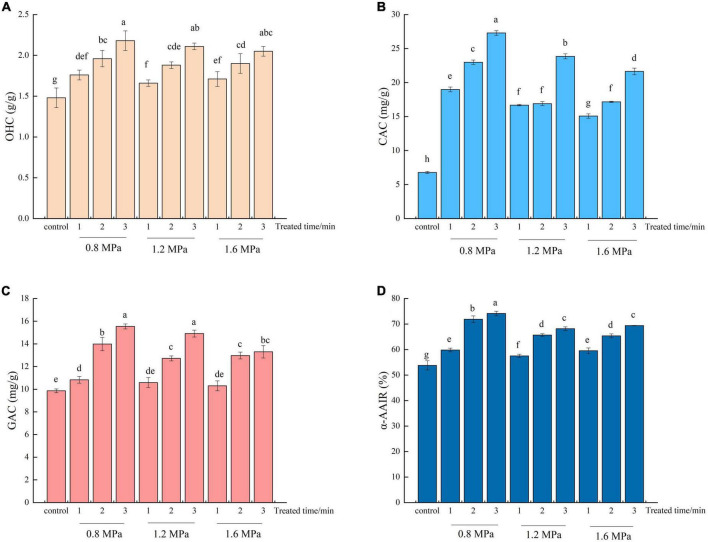
The oil holding capacity (OHC) **(A)**, cholesterol adsorption capacity (CAC) **(B)**, glucose adsorption capacity (GAC) **(C)** and α-Amylase activity inhibition ratio (α-AAIR) **(D)** of adlay bran soluble dietary fiber before and after steam explosion (SE). Different lowercase letters over column indicate significant differences (*p* < 0.05).

The OHC of SDF is an important indicator to maintain food flavor (36). As shown in Figure 2A, SE pretreatment effectively increased (*p* < 0.05) the OHC of SDF. Moreover, increasing residence time generally led to SDF with stronger OHC. The OHC of SDF treated by SE was increased from 1.48 g/g to 2.18 g/g. The maximum OHC of SDF was obtained at 0.8 MPa, 3 min, which was 1.47-fold greater than that of the control. This increase in OHC was probably caused by the increased in short-chain DF content after SE, which increased oil absorption (22). Kong et al. (37) reported that the increase in OHC for wheat bran power might be attributed to the SE-assisted superfine grinding process changing the spatial structure of the bran, with its porosity and looseness allowing the bran power to hold more oil molecules. In addition, Wang et al. (22) also reported that the spatial structure of SDF is altered by SE, with looseness and swelling properties allowing SDF to accommodate more oil molecules. Since SDF absorbs fat, increases satiety, and increases bowel motility, high OHC can help with the control of weight (38).

#### 3.3.2. CAC

Dietary fibers with high cholesterol adsorption capacities can exhibit hypolipidemic effects *in vivo* (39). CAC is an essential indicator for the evaluation the hypolipidemic performance of DFs (40). According to the results shown in Figure 2B, SE pretreatment significantly enhances cholesterol absorption capacity. The CAC of SDF increased with increasing residence time at a certain pressure. After SE processing, the CAC value significantly increased, especially for samples treated by SE at 0.8 MPa, 3 min, which reached values of up to 27.29 mg/g. Zhai et al. (41) reported that the surface of SE-SDF exhibits more binding sites, promoting adsorption. Moreover, SE disrupted the internal disordered structure of SDF, increasing the exposure of polar groups on the surface, which was more favorable to its adsorption of cholesterol (42). Kong et al. (37) also found that SE-treated wheat bran powder exhibited superior cholesterol adsorption. The high CAC of SDF from adlay bran treated by SE may indicate a high potential hypocholesterolemic effect and represent a valuable dietary resource for cholesterol-lowering foods.

### 3.4. Hypoglycemic capacity of SDF

#### 3.4.1. GAC

Glucose adsorption capacity is a significant indicator for accessing the ability of SDF to adsorb glucose (36). The GAC of SDF from adlay bran was presented in Figure 2C. SE increased significantly the GAC of SDF (except 1.2 MPa, 1 min and 1.6 MPa, 2 min). The GAC of SDF increased from 9.85 to 15.54 mg/g after SE. The GAC of SDF pretreated with SE at 0.8 MPa, 3 min was the highest, representing a significant increase of 57.77% compared to the untreated adlay bran. After SE, the specific surface area of *Rosa roxburghii* pomace SDF was increased, as were the numbers of pores and folds, which was beneficial to the adsorption of glucose molecules (41). In addition, the modified SDF surface exposed more polar and non-polar groups, which further enhanced the interaction between DF and glucose molecules (43). The results above show that SE pretreatment can enhance the GAC of adlay bran SDF, delay glucose diffusion and reduce the effective concentration of glucose, which may be helpful to inhibit the increase postprandial blood glucose (28).

#### 3.4.2. GDRI

The GDRI of SDF from adlay bran was presented in Table 2. SE significantly improved (*p* < 0.05) the GDRI of SDF from adlay bran compared to untreated control. The GDRI of SDF pretreated at 0.8 MPa for 3 min was the highest, with values of 36.57% at 30 min, 22.46% at 60 min, 23.84% at 90 min, and 18.30% at 120 min. Moreover, this decrease in GDRI values over time. The GDRI of DFs was related to microstructure and GAC (44). The enhanced GDRI of SDF following SE pretreatment might be attributed to the more porous surface structure associated with physical effects, potentially trapping glucose in the fiber network and thereby delaying the diffusion of glucose (18). In addition, SE increases the GAC of SDF, resulting in a higher GDRI. The results showed that SE can increased the GDRI of adlay bran SDF, delay glucose diffusion, which may be beneficial in delaying the absorption of glucose in the gastrointestinal tract (39).

**TABLE 2:** Effect of steam explosion (SE) pretreatment on glucose dialysis retardation index (GDRI) of adlay bran soluble dietary fiber (SDF).

Sample	Glucose dialysis retardation index			
	30 min	60 min	90 min	120 min
Control	10.56 ± 0.79^g^	6.76 ± 0.45^g^	6.17 ± 0.41^g^	5.43 ± 0.63^e^
0.8 MPa, 1 min	25.73 ± 1.41^c^	13.52 ± 0.64^d^	14.69 ± 1.25^c^	13.28 ± 0.49^b^
0.8 MPa, 2 min	28.00 ± 1.52^b^	15.95 ± 0.68^c^	17.87 ± 0.54^b^	17.11 ± 0.90^a^
0.8 MPa, 3 min	36.57 ± 2.30^a^	22.46 ± 1.60^a^	23.84 ± 2.49^a^	18.30 ± 0.69^a^
1.2 MPa, 1 min	13.72 ± 0.30^f^	11.62 ± 0.36^e^	8.51 ± 0.45^ef^	11.68 ± 0.47^bc^
1.2 MPa, 2 min	20.57 ± 0.69^e^	13.78 ± 0.67^d^	14.69 ± 1.42^c^	11.89 ± 2.08^bc^
1.2 MPa, 3 min	27.72 ± 3.84^b^	19.46 ± 0.94^b^	12.98 ± 0.37^d^	16.51 ± 0.66^a^
1.6 MPa,1 min	21.43 ± 0.69^e^	10.02 ± 0.30^ef^	7.23 ± 0.36^fg^	6.24 ± 0.97^de^
1.6 MPa, 2 min	28.88 ± 1.59^b^	8.38 ± 0.61^g^	9.57 ± 0.10^e^	8.04 ± 0.82^d^
1.6 MPa, 3 min	23.70 ± 0.80^d^	14.71 ± 1.60^cd^	11.91 ± 0.41^d^	11.27 ± 0.34^c^

Data represent the mean and standard deviation of each sample assayed in triplicate. Values with different letters in the same column indicate significant differences (*p* < 0.05). Control: untreated adlay bran SDF; adlay bran treated by SE at different steam pressures (0.8, 1.2, and 1.6 MPa) and different residence times (1, 2, and 3 min).

#### 3.4.3. α-AAIR

The effects of SE pretreatment on the α-AAIR of SDF samples were presented in Figure 2D. Compared with untreated SDF, SE pretreatment increased significantly the α-AAIR of SDF, reaching a maximum value of 74.14% after 0.8 MPa for 3 min, which was 37.80% higher than the untreated control. DFs with high GAC can significantly reduce the contact between starch andα-amylase by adsorbing starch granules, thereby reducing α-amylase activity (26). Furthermore, a high GDRI indicates that SDF can effectively delay the diffusion of produced glucose, resulting in reduced α-amylase activity (45). Liu et al. (42) concluded that SE-*R. roxburghii* pomace SDF had a higher α-AAIR due to the fact that it adsorbs and immobilizes more enzymes after SE, resulting in more α-amylase inhibition. The a-AAIR results suggest that the enhanced abilities of the SE-SDF from adlay bran to reduce amylase activity are indicative of its potential to reduce glucose levels in serum.

### 3.5. Particle size properties

The particle size distributions of SDF from untreated adlay bran and SE-treated (0.8 MPa, 3 min) adlay bran were investigated and are presented in Table 3. The D50 value of SDF from untreated adlay bran was 72.80 μm, while that of SDF with SE treatment was 51.07 μm (*p* < 0.05). After SE treatment, D (4, 3) and D (3, 2) significantly decreased from 92.07 to 69.63 μm and from 32.30 to 24.60 μm, respectively, whereas the specific surface area of the SE-treated sample increased by 1.31-fold in comparison with the untreated control. These results showed that SE loosens the surface structure of SDF and decreases the particle size, resulting in increased surface area, which corroborates reported results for wheat bran powders and dietary fiber from *R. roxburghii* pomace (37, 41). The particle size distribution affects the functionality of fiber (46). The reduction in particle size may lead to changes in physicochemical properties and biological activities of SDF (26). Dubey et al. (47) reported that plant cellulose with small particle size exhibited enhanced capacities to hold water and bind sugars. SDF from adlay bran treated with SE had a larger specific surface area, facilitates physiological functions related to substance adsorption.

**TABLE 3:** Particle size distributions of soluble dietary fiber (SDF) from adlay bran.

Sample	D_10_ (μm)	D_50_ (μm)	D_90_ (μm)	D_(3, 2)_ (μm)	D_(4, 3)_ (μm)	Specific surface area (cm^2^/g)	Span
Control	12.93 ± 0.15^a^	72.80 ± 2.26^a^	204.33 ± 5.13^a^	32.30 ± 0.46^a^	92.07 ± 1.45^a^	126.3 ± 1.87^b^	2.63 ± 0.02^b^
SE-SDF	9.71 ± 0.15^b^	51.07 ± 2.43^b^	159.67 ± 7.64^b^	24.60 ± 0.56^b^	69.63 ± 3.30^b^	165.70 ± 3.74^a^	2.93 ± 0.00^a^

Data represent the mean and standard deviation of each sample assayed in triplicate. Different lowercase letters in the same column indicate significant differences (*p* < 0.05). [Steam explosion (SE)-SDF: treated by SE at 0.8 MPa, 3 min].

### 3.6. SEM analysis

**FIGURE 3:**
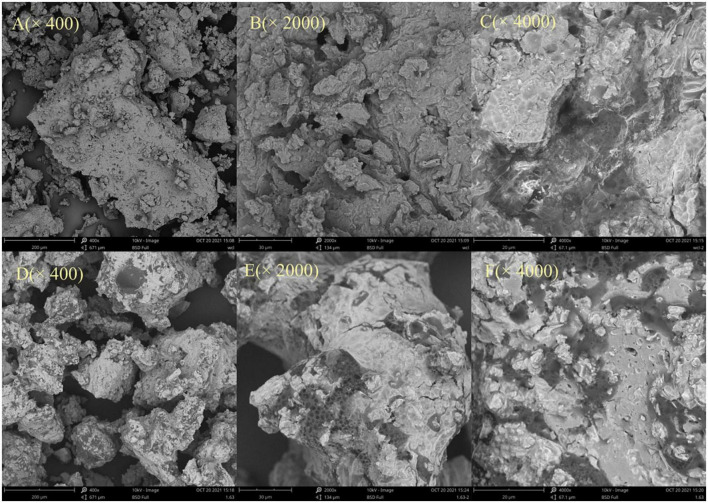
Scanning electronic micrographs of soluble dietary fiber (SDF) from untreated adlay bran **(A–C)** and SDF from steam explosion (SE)-treated adlay bran **(D–F)**. Magnifications are 400×, 2,000×, and 4,000×.

Steam explosion treatment exposes the samples to high temperature and pressure, which is the most obvious and essential feature of SE technology. The surface morphology of SDF was shown in Figure 3, the structure of the material significantly changed after SE. Specifically, the surface of SDF from untreated adlay bran was dense and intact (Figures 3A–C). After magnification 4,000 times it is clear that the 0-SDF has an overall blocky appearance (Figure 3C). In contrast, after SE pretreatment, SDF showed honeycomb-like porous with rough and wrinkled surfaces (Figures 3D–F). The SE-SDF surface showed a fluffy structure with a significant increase in internal specific surface area and porosity when the magnification was 4,000 times (Figure 3F). This is because, during SE, water vapor penetrates into the cells and tissues of the adlay bran. When the high pressure is momentarily lifted, water within the sample rapidly expands and overflows, generating a huge shear force that destroys the fiber structure (48). Gan et al. (49) found that the degradation of cellulose and hemicellulose may promote the formation of cracked honeycomb structures on the surface. After SE, the cellulose and hemicellulose contents of adlay bran decreased, resulting in a honeycomb structure of SDF.

Porosity, swelling and regional chemistry can explain some of the physiological effects of DF (21). The SEM results indicate that the dense structure of adlay bran is destroyed by SE treatment, and the size of SDF becomes smaller, in combination with the appearance of honeycomb-like porous characteristics, these changes were favorable to its functional performance. Therefore, after SE treatment, adlay bran SDF exhibited higher OHC, CAC, and GAC. Through SEM, Zhao et al. (34) found that more curls and holes appeared in sorghum after SE and making it more conducive to food fermentation. SE alters the spatial structure of okara, resulting in a significant increase in their water solubility (24). The above results show that SDF from adlay bran treated by SE can exhibit superior physicochemical and functional properties.

### 3.7. FT-IR analysis

**FIGURE 4:**
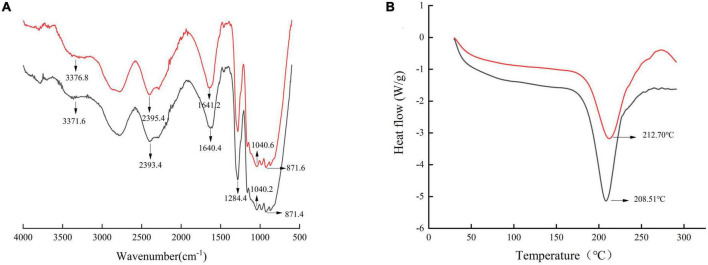
Fourier transform infrared spectroscopy (FT-IR) **(A)** and differential scanning calorimetry (DSC) thermogram **(B)** of soluble dietary fiber (SDF) from adlay bran. [Black line: untreated, red line: treated by steam explosion (SE) at 0.8 MPa, 3 min].

Fourier transformed infrared spectra of SDF were presented in Figure 4A. The broad absorption peak at 3,200–3,600 cm^–1^ was caused by O-H stretch vibrations in cellulose and semi-fiber (50). The absorption peak of SDF from SE-treated adlay bran at 3,200–3,600 cm^–1^ was broadened, indicating a stronger intermolecular hydrogen bonding force (51). The peak intensity at 3,200–3,600 cm^–1^ of the SDF after SE was higher than that of the untreated SDF, suggesting that more galacturonic acid may be present in the SDF after SE (52). The absorption peak near 1,640 cm^–1^ contained carbonyl C = O (21). The small peak at 1,200–1,400 cm^–1^ was a variable angle vibration of C-H. Moreover, the absorption peak at 1,040 cm^–1^ was attributed to C-O bond stretching in hemicellulose and lignin, suggesting the presence of sugar-aldehyde groups in SDF from adlay bran (53). The absorption peak near 871 cm^–1^ indicated a β-glycosidic bond stretching vibration of the polysaccharide molecule. The overall absorption peaks of adlay bran SDF before and after SE had similar infrared spectra; the peak shapes did not change significantly, but the positions and absorption intensities of some absorption peaks were changed.

### 3.8. Molecular weight distribution

The molecular weight distribution of SDF from untreated adlay bran and SE-treated (0.8 MPa, 3 min) adlay bran were shown in Table 4. The weight-average molecular weight of the SDF from adlay bran after SE was significantly lower than that of the untreated sample. In addition, the polydispersity value of SE-SDF was lower than that of 0-SDF. These results indicated that the molecular weight of SDF from adlay bran after SE was reduced and that the molecular weight distribution was relatively concentrated and uniformly distributed. The decrease in molecular weight may be because SE cause the degradation of macromolecular compounds to micromolecular compounds (43). In addition, SE treatment may break the chemical and hydrogen bonds in macromolecules such as cellulose and break the macromolecular crystal lattice at the molecular level (19). Lower Molecular Weight of SDF usually has better functionality and bioactivity (54). The lower molecular weight indicates better solubility and lower viscosity of SDF (55).

**TABLE 4:** Molecular weight of soluble dietary fiber (SDF) from untreated and steam explosion (SE)-treated adlay bran.

Sample	Number average molecular weight Mn (kDa)	Weight average molecular weight Mw (kDa)	Polydispersity Pd (Mw/Mn)
Control	1,743	3,795	2.18
SE-SDF	1,709	2,991	1.75

Values are obtained through independent experiments. SE-SDF: treated by SE at 0.8 MPa, 3 min.

### 3.9. Monosaccharide composition

The monosaccharide composition of SDF from untreated adlay bran and SE-treated (0.8 MPa, 3 min) adlay bran were shown in Table 5. All the SDF samples were composed of seven monosaccharides, including mannose, rhamnose, glucose, galactose, xylose, arabinose, and fucose, and glucose was the major monosaccharide. This indicated that soluble polysaccharides and pectin were the main components of SDF. The monosaccharide species in the SDF remained unchanged after SE treatment, but differed in content. The content of fucose in SDF decreased after SE treatment, which was consistent with that reported by Zhao et al. (43). However, the mannose, glucose, galactose, xylose, and arabinose contents in SDF increased by 29.76, 15.96, 38.65, 173.33, and 86.32% after SE treatment. SE treatment degraded the cellulose, hemicellulose and lignin in adlay bran to small molecular weight oligosaccharides, thereby increasing the mannose, glucose, galactose, xylose, and arabinose content of the modified SDF (56, 57). However, some low molecular weight sugars may be further degraded to smaller molecular products, which cannot be sedimented by alcohol, resulting in a decline in their monosaccharide content. In conclusion, SE treatment changed the content of monosaccharide in SDF which may have resulted in better functional activity of SDF.

**TABLE 5:** Monosaccharide composition of soluble dietary fiber (SDF) from untreated and steam explosion (SE)-treated adlay bran.

Monosaccharide content (g/100g)	Control	SE-SDF
Mannose	3.36 ± 0.03^b^	4.36 ± 0.14^a^
Rhamnose	1.56 ± 0.01^b^	1.86 ± 0.03^a^
Glucose	16.29 ± 0.14^b^	18.89 ± 0.38^a^
Galactose	6.21 ± 0.03^b^	8.61 ± 0.16^a^
Xylose	0.75 ± 0.01^b^	2.05 ± 0.07^a^
Arabinose	1.17 ± 0.00^b^	2.18 ± 0.13^a^
Fucose	0.33 ± 0.01^a^	0.19 ± 0.02^b^

Data represent the mean and standard deviation of each sample assayed in triplicate. Different lowercase letters in the same column indicate significant differences (*p* < 0.05). (SE-SDF: treated by SE at 0.8 MPa, 3 min).

### 3.10. Thermal stability

Differential scanning calorimetry was a thermal analysis method, which can obtain the heat capacity at high temperature in a short time. DSC curves for SDF are showed in Figure 4B. All samples showed typical endothermic peaks, and the peak temperature increased from 208.51 ± 0.15 to 212.70 ± 0.25°C after SE treatment. There was a significant (*p* < 0.05) upshift in the peak temperature of the treated sample, which could be attributed to the increase in short-chain SDF in comparison with the control, because the short-chain SDF has strong hydrogen bonds within its bonding site that require more energy for structural disruption (58). This conclusion is in strong agreement with previous results (21). SE-SDF from adlay bran has better thermal stability. Ideal thermal properties are crucial to maintain the stability of key nutrients and volatiles in samples (59). Similarly, Zhai et al. and Wang et al. concluded that the thermal stability of SDF from *R. roxburghii* pomace and orange peel by SE treatment were higher than that of SDF from untreated (29–41). Karakoti et al. also demonstrated that microfiber isolated from *Hibiscus sabdariffa* var. *altissima* fiber by SE have better thermal stability (60).

## 4. Conclusion

Adlay bran is rich in DF but low in SDF, which seriously affects its nutritional value and physiological functions. Appropriate SE treatment can increase significantly the extraction rate of SDF from adlay bran. When steam strength of 0.8 MPa was applied for 3 min, SDF content increased from 7.35 to 9.37%. When the SE intensity was too high, SDF contents decreased. Compared with SDF from untreated adlay bran, the SDF from SE-treated adlay bran had improved OHC and CAC and showed better hypoglycaemic capacity. The average molecular weight of SDF decreased after SE treatment. In addition, the structure of SDF from SE-treated adlay bran was disrupted and exhibited porous, honeycomb-like features, which could contribute to changes in bioactivity and functional properties. This study shows that SE can enhance the SDF content of adlay bran and effectively improve its physicochemical properties and *in vitro* hypoglycemic activity. At the same time, this study provides new ideas for utilizing SDF from adlay bran and developing new food products.

## Data availability statement

The original contributions presented in this study are included in this article/supplementary material, further inquiries can be directed to the corresponding author.

## Author contributions

XT: data curation, methodology, formal analysis, writing – original draft, and writing – review and editing. ZW: investigation, data curation, and software. JZ: investigation and data curation. JK: funding acquisition and supervision. GC: funding acquisition and project administration. MD: funding acquisition, supervision, project administration, writing – review and editing, and validation. All authors contributed to the article and approved the submitted version.
